# Ascertainment of chronic diseases using population health data: a comparison of health administrative data and patient self-report

**DOI:** 10.1186/1471-2458-13-16

**Published:** 2013-01-09

**Authors:** Elizabeth Muggah, Erin Graves, Carol Bennett, Douglas G Manuel

**Affiliations:** 1 C.T. Lamont Primary Health Care Research Centre, Élisabeth Bruyère Research Institute, Ottawa, Ontario, Canada; 2 Department of Family Medicine, University of Ottawa, Ottawa, Ontario, Canada; 3 Ottawa Hospital Research Institute, Ottawa, Ontario, Canada; 4 Institute for Clinical Evaluative Sciences, Ottawa and Toronto, Ontario, Canada; 5 Health Analysis Division, Statistics Canada, Ottawa, Ontario, Canada; 6 Department of Family Medicine and Epidemiology and Community Medicine, University of Ottawa, Ottawa, Ontario, Canada; 7 Dalla Lana School of Public Health, University of Toronto, Toronto, Ontario, Canada

**Keywords:** Chronic disease ascertainment, Health administrative data, Chronic diseases, Population health survey

## Abstract

**Background:**

Health administrative data is increasingly being used for chronic disease surveillance. This study explored agreement between administrative and survey data for ascertainment of seven key chronic diseases, using individually linked data from a large population of individuals in Ontario, Canada.

**Methods:**

All adults who completed any one of three cycles of the Canadian Community Health Survey (2001, 2003 or 2005) and agreed to have their responses linked to provincial health administrative data were included. The sample population included 85,549 persons. Previously validated case definitions for myocardial infarction, asthma, diabetes, chronic lung disease, stroke, hypertension and congestive heart failure based on hospital and physician billing codes were used to identify cases in health administrative data and these were compared with self-report of each disease from the survey. Concordance was measured using the Kappa statistic, percent positive and negative agreement and prevalence estimates.

**Results:**

Agreement using the Kappa statistic was good or very good (kappa range: 0.66-0.80) for diabetes and hypertension, moderate for myocardial infarction and asthma and poor or fair (kappa range: 0.29-0.36) for stroke, congestive heart failure and COPD. Prevalence was higher in health administrative data for all diseases except stroke and myocardial infarction. Health Utilities Index scores were higher for cases identified by health administrative data compared with self-reported data for some chronic diseases (acute myocardial infarction, stroke, heart failure), suggesting that administrative data may pick up less severe cases.

**Conclusions:**

In the general population, discordance between self-report and administrative data was large for many chronic diseases, particularly disease with low prevalence, and differences were not easily explained by individual and disease characteristics.

## Background

In the past decade chronic diseases have emerged as the most important health and health care problem in developed countries [1]. Accurate ascertainment of chronic disease is essential for sound research, clinical care and health care planning. While multiple data sources have been used to identify persons with chronic diseases—including population health surveys, disease registries, medical chart abstraction, and administrative databases—no method has emerged as the gold standard for diagnosis. Increasingly, health administrative data is being used for disease ascertainment and surveillance. While administrative data is not collected for the purposes of disease surveillance and there are concerns about the accuracy of the diagnostic information, it is a relatively inexpensive method that can provide information on large populations and can be explored overtime. In addition, administrative data can often be compared directly with other data sources, such as self-reported surveys, using individual record linkage techniques. Regardless of the data source used, there will be missed cases. The quality of the research using these data will depend on the identification, measurement of these errors, and correction or discussion of biased results.

Research comparing disease ascertainment using health administrative data with other data sources has found that agreement between sources relies on many factors, including individual and disease characteristics and the specific methods applied to the data [2-6]. Most published research has been case ascertainment validation studies of disease definitions applied to administrative data to identify disease cases [3,4,7,8]. Only a few studies have explored the accuracy of administrative data compared with self-report of chronic conditions across chronic conditions [9,10]. Agreement between these sources is higher for chronic diseases that are well-defined and require ongoing management, such as diabetes, and is lower for poorly defined diseases such as congestive heart failure [9,10]. Worsening comorbidity, measured by number of chronic conditions, appears to be associated with lower agreement—particularly for those diseases where agreement is already poor [11,12].

The objective of this study is to describe the validity of administrative data compared with self-reported disease status for the ascertainment of seven common chronic conditions in the general population and to explore the relationship with self-reported disease burden. This research takes advantage of individually linked health administrative and population survey data available for the population of Ontario, the largest province in Canada.

Our research expands on what is known from previous validation studies of administrative data by using the validated disease algorithms to identify cases in the Ontario provincial health administrative data and compares prevalence estimates with the self-report of chronic diseases in the Canadian Community Health Survey (CCHS). A limitation of previous studies was that they relied on typical measures of concordance such as Kappa scores or sensitivity and specificity to determine concordance; but, in the absence of a gold standard data source and when the prevalence of a condition is particularly high or low, these measures may not be as accurate and the underlying patterns in the data are obscured. This study will present a range of concordance measures to explore the validity of administrative data for disease ascertainment. A number of diseases are examined to explore whether concordance is similar across conditions. Finally, while previous research has used a count of chronic conditions to measure morbidity, we explore the relationship between morbidity and concordance of the data with the Health Utilities Index (HUI) [13]. To our knowledge, this is the first time that the HUI has been used to understand concordance in disease ascertainment.

## Methods

This study was conducted using population based data from the province of Ontario, Canada—with a 2011 population of more than 13 million [14]. The Institute for Clinical Evaluative Sciences (ICES) houses the health administrative data on hospital and physician billings, provided by the provincial health ministry, as well as population survey data. These data have been individually linked using an anonymized identification number.

### Population

The sample population included all adults, aged 20 years and older, with a valid Ontario health card who completed the CCHS in 2001, 2003 or 2005, and agreed to have their survey responses linked to their provincial health administrative data. Residents are eligible for the provincial health coverage if they are Canadian citizens, landed immigrants or convention refugees, make their permanent and principal home in Ontario, and are physically present in Ontario 153 days in any 12-month period. A detailed flow chart of this method is included in Additional file 1: Appendix A.

The CCHS survey uses a multi-stage, stratified, clustered design. The survey uses a probability sample and a weighting system that represents approximately 98% of the community dwelling Canadian population aged 12 years and over. Individuals living on Indian Reserves, institutional residents, full-time members of the Canadian Armed Forces, and residents of certain remote regions are excluded from the CCHS. Further details about the methods for the CCHS are reported elsewhere [15].

### Chronic disease ascertainment

#### Health administrative data

We relied on pre-existing validated case definitions, created using Ontario hospital and physician billing codes, for the following seven chronic diseases: diabetes, congestive heart failure, myocardial infarction, stroke, hypertension, asthma, chronic obstructive lung disease (COPD) [7,8,16-19]. The technical case definitions we used are presented in Additional file 1: Appendix B.

#### Canadian community health survey

The CCHS provides cross sectional estimates of health status, health determinants and health system use for Canadians. The survey asks respondents to identify if they have any one of a list of chronic health conditions which are defined as “long-term conditions that have lasted or are expected to last six months or more and that have been diagnosed by a health professional”. The relevant questions from the survey are included in Additional file 1: Appendix C.

#### Chronic disease prevalence

We calculated chronic disease prevalence in the sample population using both self-reported data and administrative definitions. For self-reported prevalence, all three cycles of the CCHS were used to identify the total number of prevalent cases. For health administrative data disease prevalence, case ascertainment was restricted to the time of the administration of the CCHS survey or earlier. Raw counts were presented for each disease in 2 × 2 tables.

#### Health utilities index

The self-reported health burden was measured using the Health Utilities Index (HUI) [20]. The HUI is a preference-based, multi-attribute health classification system that estimates a summary value of individual health where 0 = “dead” and 1 = “perfect health”. Each respondent answers questions pertaining to eight attributes of functional health: vision, hearing, speech, mobility, dexterity, emotional state, cognition and level of pain and discomfort and these attributes are assigned individual utility weights and then combined to create a summary value. HUI values were only available for CCHS cycle 1.1 respondents. The HUI values were standardized for age and sex against the 1991 Canadian population.

#### Analysis

Using the sample survey weights developed by Statistics Canada, weighted prevalence estimates were calculated to determine the total burden of each disease in Ontario in the study time period. Population prevalence was calculated by dividing the total weighted number of cases (separately done for self reported and health administrative definitions) by the total weighted population of the cohort.

The measures of concordance presented included: sensitivity (of self-report), specificity (of self-report), proportion of positive and negative agreement and Cohen’s kappa coefficient. Details on the calculation of these measures are included in Additional file 1: Appendix D. The kappa coefficient, a widely used as a measure of agreement between raters, indicates the proportion of agreement beyond that expected by chance. Levels of agreement for kappa were considered to be poor (*κ* < 0.20), fair (*κ* = 0.20 to 0.39), moderate (*κ* = 0.40 to 0.59), good (*κ* = 0.60 to 0.79), or very good (*κ* = 0.80 to 1.00) [4,21].

We calculated the median and interquartile range (25^th^ percentile- 75^th^ percentile) for the Health Utility Index (HUI) only for participants in cycle 1.1 of CCHS.

All calculations were performed using SAS 9.2.

## Results

The total sample included 99,108 respondents of which 85,549 were aged 20 years or older. Characteristics of the sample population, weighted to reflect the general Ontario population, are presented in Table 1. Those aged 65 years and older made up 16.1% of the population and 34.3% of the population had one or more chronic conditions.

**Table 1:** **Demographic and clinical characteristics of Ontarians who completed the Canadian Community Health Survey **(**Cycle 1**.**1**, **2**.**1**, **or 3**.**1**) **and agreed to link to health administrative data**, **N** = **85**,**549**

**Characteristic**	**Sample size**	Represented population*	**% (totals for each variable sum to 100%)**
Sex			
Male	38743	12 900 000	48.8%
Female	46806	13 500 000	51.2%
Age Group (years)			
20-44	36429	13 600 000	51.3%
45-64	28508	8 620 000	32.6%
65-74	11570	2 520 000	9.5%
75+	9042	1 730 000	6.6%
Income Quintile			
1 (lowest)	17396	4 850 000	18.4%
2	17361	5 110 000	19.3%
3	17247	5 370 000	20.3%
4	17215	5 580 000	21.1%
5 (highest)	16126	5 490 000	20.8%
Missing	204	29 000	0.1%
Number of Chronic Diseases (based on health administrative data)			
0	50942	17 370 000	65.7%
1	22178	6 170 000	23.3%
2	8249	2 000 000	7.6%
3	2856	625 000	2.4%
4	979	196 000	0.7%
5	270	55 000	0.2%
6+	75	14 300	0.1%

*Population estimated using the Canadian Community Health Survey sampling weights.

The unweighted counts, prevalence, and concordance measures are presented in Table 2. Kappa statistics demonstrated good or very good agreement for hypertension and diabetes, moderate agreement for myocardial infarction and asthma and poor or fair agreement for stroke, congestive heart failure and COPD. The highest agreement was for diabetes (Kappa 0.8). Percent positive agreement was much lower than negative agreement across all diseases. For example, positive agreement was 34% for COPD and 37% for stroke while percent negative agreement was 94% and 99%, respectively.

**Table 2:** **Unweighted prevalence and concordance measures for chronic diseases using health administrative and self**-**reported data**

**Self**-**reported data**	**Health administrative data**				
**Diabetes**	Yes	No	Total	Kappa	0.80 (0.80, 0.81)
Yes	5,312	474	5,786	Sensitivity of self-report	0.73
No	1,867	77,848	79,715	Specificity of self-report	0.99
Total	7,179	78,322	85,501	Positive agreement	82%
(missing = 48)				Negative agreement	99%
				Prevalence HA vs. SR	8.4% vs. 6.8%
**Stroke**	Yes	No	Total		
Yes	433	1,020	1,453	Kappa	0.36 (0.34, 0.39)
No	453	83,592	84,045	Sensitivity of self-report	0.49
Total	886	84,612	85,498	Specificity of self-report	0.99
(missing = 51)				Positive agreement	37%
				Negative agreement	99%
				Prevalence HA vs. SR	1.0% vs. 1.7%
**Hypertension**	Yes	No	Total		
Yes	15,314	2,530	17,844	Kappa	0.66 (0.65, 0.66)
No	8,284	59,293	67,577	Sensitivity of self-report	0.65
Total	23,598	61,823	85,421	Specificity of self-report	0.96
(missing = 128)				Positive agreement	74%
				Negative agreement	92%
				Prevalence HA vs. SR	27.6% vs. 20.8%
AMI*	Yes	No	Total		
Yes	732	1,326	2,058	Kappa	0.48 (0.45, 0.50)
No	213	54,799	55,012	Sensitivity of self-report	0.77
Total	945	56,125	57,070	Specificity of self-report	0.98
(missing = 74)				Positive agreement	49%
				Negative agreement	99%
				Prevalence HA vs. SR	1.7% vs. 3.6%
CHF*	Yes	No	Total		
Yes	413	458	871	Kappa	0.33 (0.30, 0.35)
No	1,151	55,042	56,193	Sensitivity of self-report	0.26
Total	1,564	55,500	57,064	Specificity of self-report	0.99
(missing = 80)				Positive agreement	34%
				Negative agreement	99%
				Prevalence HA vs. SR	2.7% vs. 1.5%
**Asthma**	Yes	No	Total		
Yes	4,620	2,742	7,362	Kappa	0.55 (0.54, 0.56)
No	3,730	74,417	78,147	Sensitivity of self-report	0.55
Total	8,350	77,159	85,509	Specificity of self-report	0.96
(missing = 40)				Positive agreement	59%
				Negative agreement	96%
				Prevalence HA vs. SR	9.8% vs. 8.6%
**COPD** (**≥ 35 years**)	Yes	No	Total		
Yes	1,880	1,842	3,722	Kappa	0.29 (0.27, 0.30)
No	5,474	56,916	62,390	Sensitivity of self-report	0.26
Total	7,354	58,758	66,112	Specificity of self-report	0.97
(missing = 12)				Positive agreement	34%
				Negative agreement	94%
				Prevalence HA vs. SR	11.1% vs. 5.6%

*For CCHS cycle 1.1 and 2.1 — assumes ‘no’ to heart disease also means ‘no’ to AMI or CHF.

Abbreviations: AMI = acute myocardial infarction; CHF = congestive heart failure; COPD = chronic obstructive pulmonary disease; HA = health administrative data; SR = self-reported data.

Weighted prevalence by self-reported data was lower than prevalence by administrative data for all diseases except stroke and acute myocardial infarction (Table 3). Prevalence estimates ranged from 0.7% (stroke) to 22.1% (hypertension) for administrative data and from 1.1% (congestive heart failure) to 16.7% (hypertension) for self-reported data. The relative difference in prevalence estimated by the two methods was greatest for acute myocardial infarction, COPD, and stroke, while the relative estimates for asthma, hypertension, and diabetes were closer.

**Table 3:** **Prevalence of selected chronic diseases**, **Ontario 2001**-**2005**

	Weighted prevalence N (%)*		
	**Health administrative data**	**Self**-**reported data**	**Missing(self-reported)**
COPD	1 560 000 (8.1)	821 000 (4.3)	3 390 (0.0)
Asthma	2 530 000 (9.6)	2 070 000 (7.8)	7 380 (0.0)
CHF	312 000 (1.8)	185 000 (1.1)	18 200 (0.1)
AMI	221 000 (1.3)	449 000 (2.6)	14 200 (0.1)
Hypertension	5 840 000 (22.1)	4 410 000 (16.7)	39 500 (0.2)
Diabetes	1 890 000 (7.2)	1 440 000 (5.4)	12 700 (0.1)
Stroke	177 000 (0.7)	333 000 (1.3)	3 390 (0.0)

*Population estimated using the Canadian Community Health Survey sampling weights.

Abbreviations: AMI = acute myocardial infarction; CHF = congestive heart failure; COPD = chronic obstructive pulmonary disease.

The standardized HUI scores ranged from 0.64 for persons self-reporting with stroke to 0.93 for persons with asthma identified on either self-report or administrative data. Self-reported cases of chronic disease had lower median HUI scores for stroke, congestive heart failure and acute myocardial infarction (Table 4). For all other conditions cases identified by self-report and health administrative data had similar HUI scores.

**Table 4:** **Health Utilities Index** (**HUI**) **for cases identified by self**-**report and health administrative data**, **standardized by age and sex**

	**Health administrative data**		**Self**-**reported data**	
	**N**	**Median (IQR)**	**N**	**Median (IQR)**
COPD	2,265	0.87 (0.61,0.97)	1,326	0.84 (0.56,0.97)
Asthma	2,535	0.93 (0.77,0.97)	2,452	0.93 (0.74,0.97)
CHF	743	0.78 (0.37,0.92)	444	0.66 (0.33,0.91)
AMI	430	0.91 (0.63,0.97)	946	0.83 (0.47,0.97)
Hypertension	6,860	0.91 (0.72,0.97)	5,143	0.91 (0.69,0.97)
Diabetes	2,038	0.91 (0.61,0.97)	1,672	0.91 (0.60,0.97)
Stroke	268	0.78 (0.42,0.92)	444	0.64 (0.29,0.87)

Abbreviations: AMI = acute myocardial infarction; CHF = congestive heart failure; COPD = chronic obstructive pulmonary disease; IQR = interquartile range.

## Discussion

We evaluated agreement between health administrative data and self-report for ascertainment of chronic disease, in a population of community dwelling Ontarian residents, using linked population-based data. With the exception of acute myocardial infarction and stroke, prevalence estimates for diseases were higher based on health administrative data compared to self-report data. In general, we found that there was a good level of agreement between data sources only for diabetes and hypertension. For the remaining diseases that were examined, there was considerable discordance in ascertainment that could only be partially explained by individual and disease characteristics. There are likely multiple reasons for these discordances that include: disease specific biases, misclassification due to the disease definitions used and the prevalence of the disease.

Okura proposes that diseases which are less familiar to patients and have nonspecific and intermittent symptoms, such as heart failure or chronic lung disease, may be particularly prone to underreporting by patients [12]. Conversely, administrative data may be more likely to identify chronic diseases requiring ongoing contact with the health care system [10,12]. This is in keeping with our results—where disease prevalence, by health administrative data, was higher for most diseases. Our finding that the self-reported prevalence of stroke or myocardial infarction was higher than the prevalence from administrative data is also consistent with other studies [11,12,22]. These two diseases are commonly known in the community and this may lead to patients falsely attributing their symptoms to them. False-positive rates of self-reported stroke ranging from 5% to 15% have even been reported from specialized stroke units, mostly from patients admitted with transient ischaemic attacks [23]. Rosamond et al. found a 40% false-positive self-report of myocardial infarction among patients in a coronary care unit, primarily due to hospitalization for unstable angina [24].

The particular question used in a survey and the case definition employed in administrative data can also affect ascertainment. In general, health administrative definitions restrict to patients with hospitalization or repeated health care contact for a disease and have a limited look-back period. This could lead to underreporting by administrative data particularly important for “event-based” diseases such as stroke and myocardial infarction where “silent” events not requiring hospitalization or events that occur outside the time period are not identified. The particular question used for ascertainment can impact ascertainment. For example, in this study the survey question for stroke was “do you suffer from the consequences of a stroke” and the health administrative data definition identified all persons admitted to the hospital with a diagnosis of stroke or transient ischemic attack (Additional file 1: Appendices B and C).

There is no agreement about which concordance measure is most valid when comparing ascertainment between data sources. Level of agreement in this study varied widely depending on the measure used particularly for the low prevalent diseases. For example, while stroke concordance was very high when comparing raw prevalence estimates (1.0% and 1.7% for administrative and self-report) it was only fair according to kappa (κ = 0.36). Some concordance measures have known limitations that are important in this context: sensitivity and specificity are less valid when no gold standard for diagnosis exists and the Kappa statistic is unreliable in the setting of a significant imbalance in the 2 × 2 table [21]. In a recent review evaluating the quality of health administrative data, Benchimol et al. proposed that a minimum of four statistical measures should be used to assess for accuracy and validity of administrative data source to help mitigate these limitations [25]. Others have similarly recommended that when measuring agreement in administrative data researchers should report kappa, the prevalence, positive agreement, negative agreement and relative frequency of each cell (a, b, c and d) [26]. While there are other measures of agreement, such as the prevalence adjusted Kappa, these may not be as accurate in the setting of low prevalent conditions. We agree with these general guidelines, and we found that looking at the raw counts in a 2 × 2 table often revealed most clearly the patterns of discordance in a particular disease. Until the patterns of concordance for specific diseases are more clearly understood, using summary concordance measures (including prevalence estimates) alone may obscure the underlying patterns and should be avoided. In addition the measures selected for concordance will need to consider the particularities of the disease and population sampled.

We were particularly interested in the relationship between morbidity and agreement. This study is the first to present and explore the relationship of HUI, a validated self-reported measure of overall disease burden, to disease ascertainment. As anticipated, we found for some conditions (myocardial infarction, stroke and heart failure) cases identified by administrative data had higher median HUI scores (thus lower reported morbidity) compared with self-report cases across all diseases. For these conditions health administrative data therefore tended to identify healthier patients than those found through self-report. While the HUI is an overall measure of morbidity, and not a disease-specific measure the severity, it is probable that the severity of underlying diseases relates strongly to overall morbidity. Our finding underscores the need for researchers to consider the clinical significance of cases identified by different data sources.

Stroke and congestive heart failure had the lowest HUI scores, the largest differences in median HUI scores, and poor concordance for the two disease ascertainment methods; while diabetes and hypertension had high HUI scores and high concordance in both median HUI and disease ascertainment. Previous research has, in general, found comorbidity is associated with poorer agreement in ascertainment [10-12]. Our findings confirm that care should be taken in the interpretation of disease estimates in population with high levels of disease burden.

### Limitations

We acknowledge that there is no clear reference standard for the ascertainment of chronic diseases. While clinical charts are often used to assess ascertainment accuracy, even this approach is not a gold standard. For example, clinic chart review for diabetes can miss cases that are not receiving glucose lowering medications, are not regular clinic attendees or who have their diabetes care provided by practitioners [17]. In our view, disease ascertainment is usually linked to disease severity, with less severe disease often poorly ascertained. Therefore, case ascertainment, the likelihood of truly being diagnosed with a disease and disease severity, health burden from disease are all intertwined. The paucity of disease-specific severity measures that use administrative data reveals an important gap in knowledge in our efforts to improve accurate ascertainment diseases using population based data.

This study excludes a number of key chronic diseases for which we do not yet have validated algorithms, but we do not feel that this affects the implications of our findings. It is clear that the relationships between ascertainment, disease, and patient characteristics are complex. Future analysis should consider multivariate methods to explore the effect of these factors.

## Conclusions

Population based data is a powerful tool for chronic disease surveillance. This study explored agreement between administrative and survey data for ascertainment of seven key chronic diseases. We found that discordance was large for many chronic diseases, particularly disease with low prevalence and that these differences were not easily explained by individual and disease characteristics. In general health administrative data tended to identify patients who were healthier, although we were not able to comment on if their disease specific morbidity was also lower. We find that the accuracy, validity and generalizeability of chronic disease case ascertainment methods depends on the data source used. Researchers should be mindful as to the implication of data source on their results.

## Competing interests

The authors declare there are no competing interests.

## Authors’ contributions

**EM** contributed to the concept of the study and oversaw its implementation, helped guide the analysis, was the primary author and approved the final version of the manuscript. **EG** was responsible for the data analysis, participated in the editing of the manuscript and approved the final version of the manuscript. **CB** contributed to the implementation of the study, participated in the writing of the manuscript, and approved the final version of the manuscript. **DM** contributed to the concept of the study, oversaw its implementation, helped guide the analysis and participated in the writing and approved the final version of the manuscript. All authors read and approved the final version of the manuscript.

## Authors’ information

**EM** is a new investigator at the C.T. Lamont Primary Health Care Research Centre and Bruyere Research Institute, clinician scientist at the Department of Family Medicine University of Ottawa Canada and a research fellow at the Institute for Clinical Evaluative Sciences. **EG** was an analyst at the Institute for Clinical Evaluative Sciences at the time that this research was completed, **CB** is a Research Coordinator at Institute for Clinical Evaluative Sciences, **DM** is a Senior Scientist, Ottawa Hospital Research Institute, Adjunct Scientist, Institute for Clinical Evaluative Sciences, Chair in Applied Public Health Sciences, CIHR/PHAC, Associate Professor, University of Ottawa and University of Toronto, and Associate Scientist, C.T. Lamont Primary Health Care Research Centre and Bruyère Research Institute.

## Pre-publication history

The pre-publication history for this paper can be accessed here:

http://www.biomedcentral.com/1471-2458/13/16/prepub

## Supplementary Material

Additional file 1 **Appendices. Appendix A. **Canadian Community Health Survey (CCHS) respondents from Ontario individually linked to provincial health administrative data. **Appendix B. **Technical case ascertainment definitions used for the Institute of Clinical Evaluative Sciences' (ICES) Multiple Chronic Disease Database. **Appendix C. **Canadian Community Health Survey (CCHS) questions for chronic disease case ascertainment. **Appendix D. **Calculation of concordance measures. Click here for file
