# Supplementary material for: Ascertainment of chronic diseases using population health data: a comparison of health administrative data and patient self-report
Source: BMC Public Health. 2013 Jan 9;13:16. doi: 10.1186/1471-2458-13-16 (PMC3557162; doi:10.1186/1471-2458-13-16)
Supplement: Additional file 1 — Appendices. Appendix A. Canadian Community Health Survey (CCHS) respondents from Ontario individually linked to provincial health administrative data. Appendix B. Technical case ascertainment definitions used for the Institute of Clinical Evaluative Sciences' (ICES) Multiple Chronic Disease Database. Appendix C. Canadian Community Health Survey (CCHS) questions for chronic disease case ascertainment. Appendix D. Calculation of concordance measures. [file 1471-2458-13-16-S1.docx]

Appendix A. Canadian Community Health Survey (CCHS) respondents from Ontario individually linked to provincial health administrative data

**Unique Individuals:**

***99413***

(516 Duplicates Removed)

**Linked Records in All Cycles:**

***99929***

**Eligible^1^ for provincial health coverage at Survey Date:**

***99108***

(305 Ineligible Individuals Removed)

LINKED: ***32848***

*(68.6%)*

RESPONDED

***39278***

LINKED: ***33679***

*(61.8%)*

LINKED: ***33402***

*(63.9%)*

AGREED TO LINK FILE:

***37681***

AGREED TO LINK FILE:

***40507***

AGREED TO LINK FILE:

***39486***

RESPONDED

***42777***

RESPONDED

***41766***

LINKED DATA

**CCHS Cycle 1.1 (2001)**

***47900***

**CCHS Cycle 2.1 (2003)**

***54493***

**CCHS Cycle 3.1 (2005)**

***52242***

Canadian Community Health Survey (CCHS)

1. Residents are eligible for provincial health coverage if they are Canadian citizens, landed immigrants or convention refugees, make their permanent and principal home in Ontario, and are physically present in Ontario 153 days in any 12-month period.

**Appendices: Ascertainment of chronic diseases using population health data: a comparison of health administrative data and patient self-report**

Appendix A. Canadian Community Health Survey (CCHS) Data respondents from Ontario linked to provincial health administrative data.

Appendix B. Technical case ascertainment definitions used for the Institute of Clinical Evaluative Sciences' (ICES) Multiple Chronic Disease Database.

| **Standard Exclusions Applied to All Diseases:** | |
| --- | --- |
| - Valid unique identification number/ICES Key Number (IKN) | |
| - Must be present in Registered Persons Database | |
| - Dead prior to April 1^st^ 1991 | |
| - Dead before diagnosis date | |
| - Born after diagnosis date | |
| - Must live in Ontario | |
| - Valid sex (Male, Female) in Registered Persons Database | |
| **Databases included with relevant International Classification of Diseases (ICD) Coding** | |
| - Ontario Provincial Health Insurance Plan (OHIP), ICD-9 | |
| - National Ambulatory Case Reporting System (NACRS), ICD-10-Canada   (NACRS includes data for Emergency Department (ED) and Same Day Surgery (SDS) ) | |
| - Hospital Discharge Abstract Database (DAD) (includes only acute inpatient and day surgery records) , ICD-9 prior to 2002; ICD-10-Canada from 2002 to present | |
| - Ontario Mental Health Reporting System (OMHRS), ICD-10-Canada | |
| **Asthma (ICES Derived Asthma Cohort)(1)** | |
| **Time period** | July 1991- present |
| **Case Ascertainment** | - 1 hospital admission with an asthma diagnosis code (any occurrence of the code in the DAD) **AND/OR** - 2 OHIP claims with an asthma diagnosis code in 2 years |
| **Washout Period** | 5 years |
| **Sensitivity/ Specificity** | For 0-17 year olds: 89%/72%  For 18+: 84%/76% |
| **Data Sources** | OHIP (July 1991 forward)  DAD/SDS (April 1991 forward)  NACRS (SDS only) (F2003-04 forward) |
| **Codes Used** | ICD-9: 493 (any type)  ICD-10-CA: J45, J46 (any type)  OHIP diagnosis code: 493 |
| **Notes** | Prevalence starts in 1993 (b/c need 2 years to ascertain a case)  Incidence starts in 1996  Must live in Ontario at prevalent start date |
| **Congestive Heart Failure (CHF)(ICES Derived Cohort)** (2) | |
| **Time period** | July 1991- present |
| **Case Ascertainment** | - 1 hospital admission with a CHF diagnosis code (any occurrence of the code in the DAD) **OR** - 1 OHIP/NACRS record with a CHF diagnosis code, followed within 2 years by another OHIP/NACRS record or a hospital admission with a CHF diagnosis code (any occurrence of the code in the DAD) |
| **Washout Period** | 3 years |
| **Sensitivity/ Specificity** | 84.8%/97.0% |
| **Data Sources** | OHIP (July 1991 forward)  DAD/SDS (April 1991 forward)  NACRS (SDS only) (F2003-04 forward)  NACRS (ED only) (F2002-03 forward)  OHMRS (October 2005 forward) |
| **Codes Used** | ICD-9: 428 (any type)  ICD-10-CA: I50.0, I50.1, I50.9 (any type)  OHIP diagnosis code: 428 (only 1 rec per person per service date) |
| **Ontario Myocardial Infarction Database (OMID)(ICES Derived Cohort)(3)** | |
| **Time period** | April 1991- present |
| **Case Ascertainment** | - 1 hospital admission with an AMI diagnosis code (Most respondible diagnosis only) **AND** - **NO** other AMI diagnosis code with a type 2 on same record **AND** - *None of the exclusion criteria listed below are applicable.* |
| **Washout Period** | 1 Year |
| **Sensitivity/ Specificity** | N/A |
| **Data Sources** | DAD (April 1991 forward) |
| **Codes Used** | ICD-9: 410  ICD-10-CA: I21 |
| **Notes** | *Exclusion criteria applied to cohort:*   - Non-first visit within an episode of care - Missing Year - Invalid gender, i.e. SEX ≠ M or F - Non-acute care hospital admission - Non-Ontario resident by looking at the 1^st^ two digits of RESCODE - Age < 20 or Age > 105 - Transfer from another acute care hospital - Patient signed out or Discharge with a LOS < 3 days **and** patient was **NOT** transferred **to** another acute care hospital - Non-valid Health Card number, i.e. invalid IKN - The **main** doctor service code was one of these:  \| ICD-9:   - - 30 General Surgery,   - 32 Neurosurgery,   - 33 Oral Surgery,   - 34 Orthopaedic Surgery,   - 35 Plastic Surgery,   - 36 Thoracic Surgery,   - 37 Transplant Surg,   - 38 Unknown and reference can’t not located   - 39 Urology,   - 50 Obstetrician & Gynaecologist,   - 60 Otolaryngology,   - 62 Ophthalmology,   - 64 Psychiatry,   - 87 Dentistry, or - 95 Unknown and reference can’t not located \| ICD-10-CA:   - 00030 General Surgery, - 00032 Neurosurgery, - 01003 Oral Surgery, - 00034 Orthopaedic Surgery, - 00035 Plastic Surgery, - 00036 Thoracic Surgery, - 00037 Transplant Surg, - 00039 Urology, - 00050 Obstetrician & Gynaecologist, - 00060 Otolaryngology, - 00062 Ophthalmology, - 00064 Psychiatry, - 00073 General Surgical Oncology, - 01000 Dentistry Group, - 01001 Dentist, - 01002 Dental Surgeon, - 01004 Orthodontist, - 01005 Paedodontist, - 01006 Periodontist, - 01007 Oral Pathologist, - 01008 Endodontist, - 01009 Oral Pathologist, - 01010 Dental Hygienist/Assistant, or - 01011 dental Mechanic \| \| --- \| --- \| |
| **Ontario Diabetes Database (ODD)(ICES Derived Cohort)(4;5)** | |
| **Time period** | July 1991- present |
| **Case Ascertainment (Pediatric eg < 19 yrs old)** | - 4 OHIP diagnosis code claims **OR** - 1 OHIP fee code in 2 years and at least 1 OHIP claim prior to 19^th^ birth date |
| **Case Ascertainment (Bridge)** | - 1 or 2 OHIP diagnosis code claims prior to 19^th^ birth date **AND** - 1 OHIP claim after 19^th^ birth date within 2 years   *(19^th^ birth date is used as incident date)* |
| **Case Ascertainment (Adult)** | - 2 OHIP diagnosis codes **OR** - 1 OHIP fee code **OR** - 1 DAD admission after 19^th^ birth date (any occurrence of the code in the DAD) |
| **Washout Period** | None |
| **Sensitivity/ Specificity** | 86%/97%- for the original algorithm |
| **Data Sources** | OHIP (July 1991 forward)  DAD/SDS (April 1988 forward)  NACRS (SDS only) (F2003-04 forward) |
| **Codes Used** | ICD-9: 250 (any type)  ICD-10-CA: E10, E11, E13, E14 (any type)  OHIP diagnosis code: 250  OHIP fee code: Q040, K029, K030 |
| **Notes** | Gestational Diabetes records excluded  Incidence not reported for first 3 years |
| **Hypertension (ICES Derived Cohort)(6)** | |
| **Time period** | July 1988- present |
| **Case Ascertainment** | - 1 hospital admission with a hypertension diagnosis code (any occurrence of the code in the DAD) **OR** - 1 OHIP record with a hypertension diagnosis code, followed within 2 years by another OHIP record or a hospital admission with a hypertension diagnosis code (any occurrence of the code in the DAD) |
| **Washout Period** | 5 years |
| **Sensitivity/ Specificity** | 72%/95%  PPV-87%; NPV-88% |
| **Data Sources** | OHIP (July 1991 forward)  DAD/SDS (April 1988 forward)  NACRS (SDS only) (F2003-04 forward) |
| **Codes Used** | ICD-9: 401x, 402x, 403x, 404x, 405x (any type)  ICD-10-CA: I10, I11, I12, I13, I15 (any type)  OHIP diagnosis code: 401, 402, 403, 404, 405 (any type) |
| **Notes** | Gestational hypertension records excluded  Generic exclusions do not apply to this cohort |
| **Chronic Obstructive Pulmonary Disease (COPD)(ICES Derived Cohort)(1)** | |
| **Time period** | April 1988- present |
| **Case Ascertainment** | - 1 hospital admission with a COPD diagnosis code (any occurrence of the code in the DAD) **OR** - 1 OHIP record with a COPD diagnosis code |
| **Washout Period** | 6 years |
| **Sensitivity/ Specificity** | 85.0%/78.4% |
| **Data Sources** | OHIP (July 1991 forward)  DAD/SDS (April 1988 forward)  NACRS (SDS only) (F2003-04 forward) |
| **Codes Used** | ICD-9: 491, 492, 496 (any type)  ICD-10-CA: J41, J42, J43, J44 (any type)  OHIP diagnosis code: 491, 492, 496 |
| **Notes** | ***Sensitive Definition Used***  Only includes people age 35-99 years  Incidence 1994 forward |
| **Cerebral Vascular Disease (CVD)(12)** | |
| **Time period** | April 1988- present |
| **Case Ascertainment** | - 1 hospital admission with a CVD diagnosis code (any occurrence of the code in the DAD) |
| **Washout Period** | None |
| **Sensitivity/ Specificity** | N/A |
| **Data Sources** | DAD/SDS (April 1988 forward)  NACRS (SDS only) (F2003-04 forward) |
| **Codes Used** | ICD-9: 430, 431, 432, 434, 436 (any type)  ICD-10-CA: I60, I61, I62, I63, I64, G46 (any type) |
| **Peripheral Vascular Disease (PVD)(12)** | |
| **Time period** | April 1988- present |
| **Case Ascertainment** | - 1 hospital admission with a PVD intervention code (any occurrence of the code in the DAD) **AND** - Without specified diagnosis codes on the same abstract |
| **Washout Period** | None |
| **Sensitivity/ Specificity** | N/A |
| **Data Sources** | DAD/SDS (April 1988 forward)  NACRS (SDS only) (F2003-04 forward) |
| **Codes Used** | *Major:*   - CCP: 96.14, 96.15 - CCI: 1VQ93, 1VC93, 1VG93   *Minor:*   - CCP: 96.11, 96.12, 96.13 **WITHOUT**   - ICD-9: 170, 171, 213, 730, 740-759, 800-900, 901-904, 940-950 **on the abstract** - CCI: 1WL93, 1WA93, 1WE93, 1WJ93, 1WM93 **WITHOUT**   - ICD-10-CA: C40, C41, C46.1, C47, C49, D160, M46.2, M86, M87, M89.6, M90.0-M90.5, Q00, Q38-Q40, S02.0, S09.0, S15, S25, T26 **on the abstract**   *Bypass:*   - CCP: 51.25, 51.29, 50.18 **WITHOUT**   - ICD-9: 4141, 441, 442 **on the abstract** - CCI: 1KG50, 1KG57, 1KG76, 1KG35HAC1, 1KG35HHC1 **WITHOUT**   - ICD-10-CA: I67.1, I71, I72, I60, I77.0, I79.0, Q codes **on the abstract** |
| **Notes** | Definition from the undiagnosed diabetes study |
| **End Stage Renal Disease (ESRD)(12)** | |
| **Time period** | April 1988- present |
| **Case Ascertainment** | - 1 hospital admission with a ESRD diagnosis code or a kidney transplant code (any occurrence of the code in the DAD) **OR** - 1 OHIP diagnosis code or fee code for blindness, hyptertensive retinopathy, retinal photocoagulation or eye vitrectomy |
| **Washout Period** | None |
| **Sensitivity/ Specificity** | N/A |
| **Data Sources** | OHIP (July 1991 forward)  DAD/SDS (April 1988 forward)  NACRS (SDS only) (F2003-04 forward) |
| **Codes Used** | ICD-9: 584, 585, 586, 4039, 4049, 7885 (any type)  ICD-10-CA: N17, N18, N19, I12, I13, R34 (any type)  OHIP diagnosis code: 369, 362  OHIP fee code: E154, E148 |
| **Notes** | Definition from the undiagnosed diabetes study |

Appendix C. Canadian Community Health Survey questions for chronic disease case ascertainment.

| **Disease** | **Survey Question** |
| --- | --- |
| Stroke | Do you suffer from the effects of a stroke? |
| Heart Disease | Do you have heart disease? If yes then the following questions were asked:  Have you ever had a heart attack (damage to the heart muscle?)  Do you currently have angina? (chest pain or chest tightness)  Do you currently have CHF (inadequate heart beat and fluid buildup in the lungs or legs) |
| Diabetes | Do you have diabetes? |
| Asthma | Do you have asthma? |
| Chronic Obstructive Pulmonary Disease (COPD) | Do you have emphysema or chronic obstructive pulmonary disease (COPD)?" |
| Hypertension | Do you have high blood pressure? |

Appendix D. Calculation of concordance measures

| **Data source 1** | **Data source 2** | |  |
| --- | --- | --- | --- |
|  | **+** | **-** | *total* |
| **+** | **a** | **b** | a + b |
| **-** | **c** | **d** | c + d |
| *total* | a + c | b + d | N |

The values a, b, c and d denote the observed frequencies for each possible combination of disease count ascertained by the two data sources

Sensitivity: a/a+c

Specificity: d/b+d

Proportion positive agreement: 2a/N+a-d

Proportion negative agreement: 2d/N-a+d

Observed proportion of agreement (Po):a+d/N

Expected proportion of agreement (Pe):((a+c)(a+b) + (b+d)(c+d))/N^2^

Kappa: Po-Pe/1-Pe

Reference List for Appendices

(1) Gershon AS, Wang C, Guan J, Vasilevska-Ristovska J, Cicutto L, To T. Identifying individuals with physcian diagnosed COPD in health administrative databases. COPD: Journal of Chronic Obstructive Pulmonary Disease 2009;6(5):388-94.

(2) Yeung DF, Boom NK, Guo H, Lee DS, Schultz SE, Tu JV. Trends in the incidence and outcomes of heart failure in Ontario, Canada: 1997 to 2007. CMAJ 2012 Aug 1;Online early release:1-9.

(3) Tu JV, Austin P, Naylor CD, Iron K, Zhang H. Chapter 5 - Acute myocardial infarction outcomes in Ontario. 1999. Report No.: ICES Cardiovascular Atlas.

(4) Hux JE, Ivis F, Flintoft V, Bica A. Diabetes in Ontario: determination of prevalence and incidence using a validated administrative data algorithm. Diabetes Care 2002 Mar;25(3):512-6.

(5) Guttmann A, Nakhla M, Henderson M, To T, Daneman D, Cauch-Dudek K, et al. Validation of a health administrative data algorithm for assessing the epidemiology of diabetes in Canadian children. Pediatr Diabetes 2009 Jun 5.

(6) Tu K, Campbell NRC, Chen ZL, Cauch-Dudek KJ, McAlister FA. Accuracy of administrative databases in identifying patients with hypertension. Open Medicine 2007;1(1):e18.
